# Efficacy and safety assessment of a water-soluble formulation of fluralaner for treatment of natural *Ornithonyssus sylviarum* infestations in laying hens

**DOI:** 10.1186/s13071-018-2678-y

**Published:** 2018-02-20

**Authors:** Nancy C. Hinkle, Faris Jirjis, Eugene Szewczyk, Fangshi Sun, Annie Flochlay-Sigognault

**Affiliations:** 1 Department of Entomology, University of Georgia, Athens, GA 30602-2603 USA; 2 Merck Animal Health, Madison, NJ 07940-1026 USA

**Keywords:** Fluralaner, Poultry, Northern fowl mite, *Ornithonyssus sylviarum*, Efficacy

## Abstract

**Background:**

Northern fowl mite, *Ornithonyssus sylviarum* (Canestrini & Fanzago, 1877), infestations can stress birds, impairing welfare and causing substantial economic losses. A study was undertaken to determine the efficacy of an ectoparasiticide solution (fluralaner) for oral administration in the treatment of mite-infested hens.

**Methods:**

Clinically healthy, naturally mite-infested laying hens (*n* = 132), approximately 32 weeks of age, were ranked by Day -9 mite vent counts and randomized among 12 study pens, each to hold one of four treatment groups. Three groups received fluralaner-medicated water by oral gavage at dose rates of 0.25, 0.5 or 1.0 mg/kg on Days 0 and 7; one group was an untreated control (three pens for each group). Five naturally infested untreated birds were included in each pen to act as mite-infested source birds. Thus each pen, treated and control, had six non-source birds for assessment of efficacy, plus five source birds to provide ongoing challenge. Primary efficacy assessments were based on mean *O. sylviarum* vent counts from non-source birds in the control and treated group pens on Days 1, 2, 6, 8, 12, 15, 19, 22 and 26.

**Results:**

Source-birds maintained infestations throughout the study, validating the challenge to study birds. On Days 1 through 22, mean control group mite counts were significantly greater than those of the treated groups (*P* ≤ 0.013). Relative to the control group, mean *O. sylviarum* counts were reduced by at least 90% from Day 6 through Days 19, 22 and 22 in the fluralaner 0.25, 0.5 and 1.0 mg/kg groups, respectively. On Day 19, mean mite counts were lower in the 0.5 and 1.0 mg/kg groups compared with the 0.25 mg/kg group (*P* ≤ 0.018), and in the 1.0 mg/kg compared with the 0.5 mg/kg group (*P* = 0.014). There were no adverse events in treated birds.

**Conclusions:**

A fluralaner solution administered twice by gavage to laying hens with a one-week between-treatment interval was safe and effective in quickly controlling *O. sylviarum* infestations despite continuous challenge from infested birds. By eliminating mites, this fluralaner solution has the potential to improve bird health and productivity, and to eliminate the burden of topical pesticide application.

## Background

The northern fowl mite (NFM), *Ornithonyssus sylviarum* (Canestrini & Fanzago, 1877), infests a wide variety of domestic fowl and wild birds and has been described as the most important ectoparasite of poultry in the United States and as a serious pest, especially on chickens (layers, breeders) [1]. Mites are permanent ectoparasites that feed on blood, and rapid proliferation within a poultry operation can lead to heavy infestations that irritate and stress the birds, impair feed conversion efficiency, egg production and egg quality and reduce farm profitability [2–4]. Mite infestations can also be a nuisance problem in poultry-house workers [5, 6].

Current treatments involve the use of topically applied pesticides, including organophosphates, pyrethroids and carbamates [7, 8]. Application of these compounds, as dusts, sprays, high pressure sprays and mists, requires specialized machinery, is labor intensive, requires repeated treatments and even then may not produce adequate reductions in mite infestations. The existing pervasive and intractable acaricide resistance problem emphasizes the need for more convenient and effective NFM treatments [7–9].

To address that need, fluralaner has been developed as an orally administered systemic ectoparasiticide for the treatment and control of mites in laying hens. Fluralaner, an isoxazoline, is a potent inhibitor of γ-aminobutyric acid (GABA)-gated chloride channels and L-glutamate-gated chloride channels, which are widely expressed in arthropod central nervous and peripheral neuromuscular systems [10, 11]. This action of isoxazolines is different from that of any of the acaricides currently used for poultry, no resistance to the family has been recorded, and cross-resistance with any of the currently-used compounds is unlikely [11, 12]. Safety testing of repeated elevated doses of fluralaner in poultry and dogs has demonstrated that fluralaner has a wide safety margin in target species, confirming the selective action of the compound on insect and acarine nervous systems [13–15].

A study was undertaken to determine the efficacy of a drinking-water formulation of fluralaner in the treatment of hens naturally infested with *O. sylviarum*. Three dose rates were investigated, each administered by oral gavage as two single administrations, 7 days apart. By quickly killing adults and immature stages, the first dose would remove the source of mite egg production. The second dose, based on the fluralaner half-life in poultry of 5 days, would ensure sufficient duration of activity to eliminate live mites that had hatched from eggs laid prior to the first dose [16].

## Methods

This was a randomized, assessor-blinded, block-design study with an untreated control group, masked to all study personnel except the principal investigator who took no part in study assessments. The data were collected in compliance with the study protocol, the FDA/CVM Good Clinical Practice Guidance Document #85, May 9, 2001, and applicable regulatory requirements [17].

### Birds and housing

One hundred and fifty clinically healthy laying hens, ISA Browns, approximately 32 weeks of age with confirmed *O. sylviarum* infestations, were sourced from a commercial flock. To be eligible for inclusion in the study, selected birds could not have been treated with an acaricide or anthelmintic within the previous 60 days. Each bird was individually identified with metal leg bands and colored numbered leg bands coordinated with the treatments and the bird’s assigned pens. Staff performing study assessments, including mite counts, were masked to the treatment assignments corresponding to each leg band color.

Prior to randomization and allocation to treatment groups, the birds were comingled in pens. Following treatment group allocation, birds were assigned to twelve enclosed pens (1.2 × 1.8 × 1.8 m), with 11 birds per pen. The birds were held under natural lighting, initially with 11 h and 49 min of indirect daylight, declining in the next month to 10 h and 30 min, with exposure to ambient temperature and humidity. No artificial lighting was provided at night. Clean wood shavings were provided as bedding on an as-needed basis. Hens were provided ad libitum access to the standard post-peak layer diet used by the UGA Poultry Research Center, free of antibiotics, coccidiostats, anthelmintics and other medications. The feed exceeded 1994 National Research Council nutrient recommendations. Fresh feed and water were provided daily via typical poultry feeders and gravity-fed nipple waterers. Routine cleaning was performed according to standard facility practices. Birds were checked daily to ensure safety and health. No concomitant medications were administered to any of the test birds.

### Mite counts

Mites infesting hens were counted by visually inspecting the area near the vent (the cloacal opening), the area known to contain the vast majority of mites on domestic hens [18]. This process required holding the hen and carefully examining the feathers in the approximately 4 × 6 cm area anterior to the vent. The examiner parted the feathers, scrutinizing the skin and feathers to detect and count the adult stage of mites. Mite vent counts were performed on all treatment groups on Days -9 (pre-study for blocking and randomization), 1, 2, 6, 8, 12, 15, 19, 22 and 26.

### Randomization and treatment

Based on Day -9 mite vent counts, the 150 hens were ranked from highest to lowest mite numbers and the 132 hens with the highest counts were placed into 11 blocks of 12 birds (the 18 birds with the lowest mite counts were retained to be used as substitutes if needed). Within each block, SAS version 9.3 was used to randomly assign birds to the 12 study pens. Approximately 1 week prior to the first treatment day, the 12 pens were randomly allocated to either one of three fluralaner treatment groups (0.25, 0.5 or 1.0 mg/kg) or to an untreated control group, resulting in three pens per group. Following assignment, five birds from each pen, including the control pens, were randomly selected and designated as “source birds” that would remain untreated. Thus, each of the 11 pens consisted of six “non-source” birds, to be used in efficacy determination, plus five untreated mite-infested source birds. The objective of this design was to maintain blinding of study assessors, to stock the same density of birds in each pen of each group, and to ensure a consistent and similar challenge across groups for the duration of the study.

The fluralaner solution (10 mg/ml) was diluted with tap water to a concentration of 0.2 mg/ml and administered to treatment-group birds on Days 0 and 7 at dose rates of 0.25, 0.5 or 1.0 mg/kg body weight, based on weights obtained on Day -1 and Day 5 using scales checked for accuracy prior to and after each day of weighing. Dose volumes were calculated for each bird individually. The respective volume of the treatment solution was administered using a previously described avian gavage technique [19]. A 10 ml pipette was inserted via the esophagus into the crop and the appropriate volume of treatment solution dispensed before the pipette was withdrawn. Treatment solution samples were collected and sent to an analytical laboratory for determination of fluralaner concentration to compare the actual concentration to the theoretical concentration of fluralaner of 0.2 mg/ ml.

To minimize fluralaner exposure across birds in the different treatment groups, materials and equipment used were assigned to a treatment group at the start of the study. The materials for any group were then maintained separately from equipment assigned to other treatment groups.

From Day -9 through Day -1 birds were observed for general health. Following treatment on Day 0, birds were observed daily for general health and adverse events on Days 0 through 26.

### Efficacy assessment

The primary effectiveness variable was based on adult *O. sylviarum* mite counts from the six non-source birds in each of the control and fluralaner-treated group pens. The mite vent count data for each bird were transformed prior to analysis using the Y = ln(x + 1) transformation and analyzed using a mixed linear model with treatment as the fixed effect; block, pen(block) and treatment*pen(block) as the random effects. Data were analyzed on each count day separately. The primary software used for statistical calculations was SAS version 9.3.

The null hypothesis tested was that the six non-source birds in each fluralaner treatment pen would have the same mean mite counts as the six non-source birds in each control group pen, versus the alternative that the mite counts of the two groups would be different. The hypothesis was tested using a t-test at α = 0.05 (two-sided) significance level. Effectiveness was also calculated using arithmetic means.

Percent efficacy was calculated using the Abbott’s formula:

Efficacy (%) = 100 × (M_C_ - M_T_) / M_C_.

where M_C_ is the geometric mean of total vent counts of mites on untreated birds, and M_T_ is the mean number of total vent counts of mites on treated birds.

## Results

**Fig. 1:**
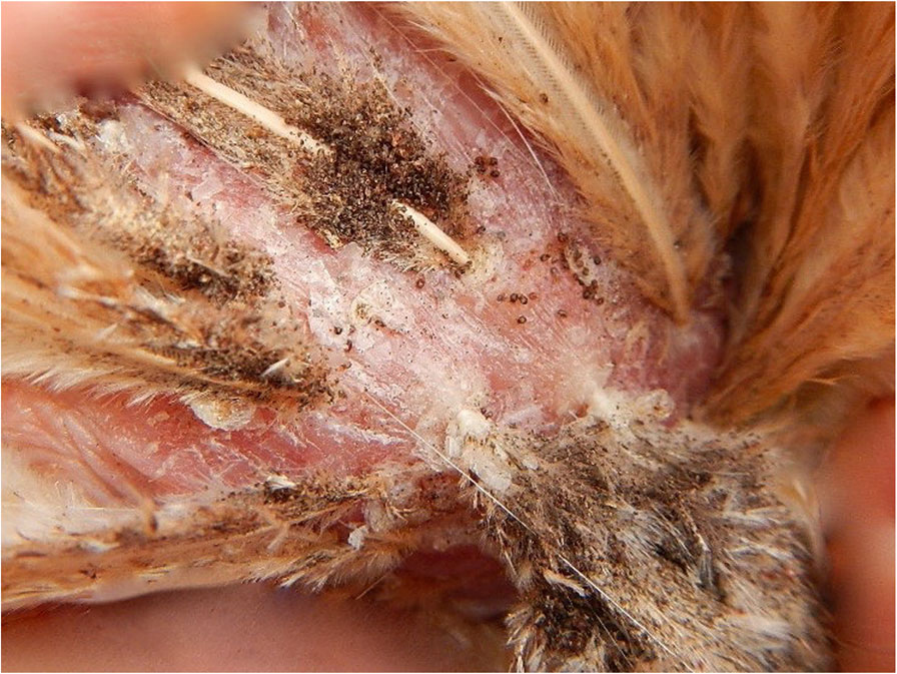
Northern fowl mites and their debris (mite feces, shed skins and eggshells) on feathers with irritated and scabbed chicken skin

The source-birds (five per pen) maintained natural mite infestation challenge throughout the study (Fig. 1, Table 1). For those penned with the control birds, geometric mean mite counts ranged from 45.3 to 76.6; for those with the fluralaner 0.25 mg/kg group from 17.0 to 51.9; for the 0.5 mg/kg group from 17.5 to 49.2; and for the 1.0 mg/kg group from 25.7 to 71.4.

**Table 1:** Geometric (arithmetic ± standard deviation) mean *Ornithonyssus sylviarum* mite vent counts in source birds that shared pens with non-source birds randomized to the treated and control groups

Day	Fluralaner (mg/kg)			
	Control	0.25	0.5	1.0
-9	74.1 (75.1 ± 86.2)	43.8 (45.1 ± 37.1)	49.2 (49.5 ± 34.1)	71.4 (74.9 ± 66.5)
8	45.3 (58.0 ± 57.1)	22.5 (23.0 ± 19.4)	22.8 (26.6 ± 37.4)	50.6 (52.8 ± 67.6)
12	49.8 (49.9 ± 38.2)	17.0 (17.0 ± 15.9)	17.5 (24.5 ± 24.1)	25.7 (26.5 ± 21.3)
15	59.1 (59.3 ± 63.7)	19.8 (20.3 ± 11.9)	31.7 (41.5 ± 53.6)	45.5 (47.2 ± 38.3)
19	76.6 (81.7 ± 64.3)	48.3 (51.8 ± 64.6)	30.4 (38.1 ± 33.7)	36.1 (37.5 ± 23.9)
22	75.1 (83.7 ± 119.1)	49.6 (55.7 ± 72.7)	35.3 (63.3 ± 163.0)	28.9 (36.1 ± 33.2)
26	48.2 (48.8 ± 31.0)	51.9 (57.5 ± 63.1)	32.5 (49.4 ± 84.4)	37.9 (39.3 ± 20.2)

Assay results of the fluralaner treatment solution showed that the average actual dose rates administered to individual birds in each of the three treated groups on Days 0 and 7 were: 0.26 and 0.24 mg/kg (for the 0.25 mg/kg group); 0.52 and 0.47 mg/kg (for the 0.50 mg/kg group); and 1.04 and 0.94 mg/kg (for the 1.0 mg/kg dose group).

On Day -9 there were no significant differences between any of the groups of non-source birds (*P* ≥ 0.532). At all assessments from Days 1 through 22 the least squares means mite counts for the control group were significantly greater than the counts for each of the fluralaner groups (Table 2). On Day 26 there were no significant differences between the control and fluralaner groups (*P* ≥ 0.085). Relative to the control group, mean *O. sylviarum* counts were reduced by at least 90% from Days 6 through 19, 22 and 22 in the fluralaner 0.25, 0.5 and 1.0 mg/kg groups, respectively (Table 3). On Day 19, the mean mite counts were significantly lower in the 1.0 mg/kg group than in both the 0.25 mg/kg (*t*-test: *t*_(66)_ = 5.0, *P* < 0.0001) and the 0.5 mg/kg groups (*t*-test: *t*_(66)_ = 2.5, *P* = 0.014), and significantly lower in the 0.5 mg/kg group than in the 0.25 mg/kg group (*t*-test: *t*_(66)_ = 2.4, *P* = 0.018). Linear and quadratic effects were both significant on Days 1, 2, 6, 8, 12, 15, 19 and 22 (*P* ≤ 0.0436).

**Table 2:** Geometric (arithmetic ± standard deviation) mean *Ornithonyssus sylviarum* mite vent counts in untreated control and in hens receiving fluralaner on Days 0 and 7

Day	Fluralaner (mg/kg)			
	Control	0.25	0.5	1.0
-9	41.5 (45.4 ± 18.4)	42.6 (49.9 ± 30.4)	44.0 (50.9 ± 27.7)	38.9 (52.4 ± 61.3)
1	32.9^a^ (41.9 ± 41.9)	12.0^b^ (13.8 ± 7.8)	12.8^b^ (15.1 ± 8.5)	12.4^b^ (13.6 ± 5.9)
2	25.2^c^ (81.8 ± 230.3)	4.3^d^ (4.9 ± 3.2)	3.1^d^ (4.0 ± 3.3)	4.3^d^ (6.7 ± 6.6)
6	24.9^a^ (37.3 ± 32.3)	0.8^b^ (1.7 ± 3.2)	0.6^b^ (1.1 ± 1.7)	0.5^b^ (0.9 ± 1.7)
8	33.9^a^ (49.2 ± 41.0)	0.2^b^ (0.5 ± 1.5)	0.1^b^ (0.1 ± 0.5)	0.3^b^ (1.1 ± 3.7)
12	25.7^a^ (49.5 ± 83.5)	0.1^b^ (0.4 ± 1.7)	0.2^b^ (0.8 ± 2.5)	0.3^b^ (2.1 ± 8.2)
15	40.4^a^ (47.6 ± 26.3)	0.4^b^ (1.0 ± 2.8)	0.7^b^ (1.4 ± 2.9)	0.4^b^ (0.8 ± 1.6)
19	50.7^a^ (56.8 ± 29.1)	4.7^b,e,g^ (8.1 ± 8.1)	1.9^b,f,k^ (3.6 ± 4.6)	0.4^b,h,m^ (1.0 ± 2.4)
22	34.6^c^ (41.6 ± 25.4)	9.4^d,e^ (10.4 ± 5.7)	3.0^d^ (7.5 ± 12.1)	1.9^d,f^ (4.1 ± 6.9)
26	27.5 (34.8 ± 22.7)	29.3 (40.4 ± 26.4)	8.2 (17.7 ± 22.6)	11.8 (14.2 ± 9.2)

Numbers within rows with a superscript are significantly different: treated group comparison with control: ^a,b^*P* < 0.0001; ^c,d^*P* < 0.05; Comparison between fluralaner groups: ^e,f^*P* < 0.05; ^g,h^*P* < 0.0001; ^k,m^*P* = 0.014. There were no significant differences between any groups on Days -9 and 26

**Table 3:** Percent reduction, compared to control group non-source birds, in geometric mean *Ornithonyssus sylviarum* mite counts of three fluralaner dose rates, each administered on Days 0 and 7

Fluralaner dose rate	Day of study								
	1	2	6	8	12	15	19	22	26
0.25 mg/kg	63.7	82.8	96.7	99.4	99.5	99.0	90.8	72.9	0.0
0.5 mg/kg	61.0	87.7	97.7	99.8	99.0	98.4	96.2	91.4	70.3
1.0 mg/kg	62.5	82.8	98.2	99.1	98.8	99.0	99.1	94.6	57.2

There was one post-treatment abnormal health observation (a leg laceration) in a source bird in the control group that was recorded as an adverse event. There were no abnormal health observations in treated birds.

## Discussion

The potent effect of fluralaner on mites has been demonstrated in dogs, applied either orally or topically, with a single treatment demonstrating complete (100%) elimination of *Sarcoptes scabiei* [20, 21]. In this chicken study, the two administrations of fluralaner at 0.5 mg/kg and 1.0 mg/kg were more than 96% effective from Days 6 through 19. By including the mite-infested source birds with treated birds, the study design created a worst-case scenario in which a high-level challenge was sustained throughout the study. Given previous investigations demonstrating complete efficacy of fluralaner against mite infestations in poultry and in dogs, we would expect that had all the birds in each pen been treated, all would have become mite-free, and the elimination of an ongoing challenge would remove the need for additional treatments [21, 22].

Following treatment, early onset of action was observed at all fluralaner dose rates, with statistically significant reductions in mite vent counts, relative to the control group, observed in all treated groups from Day 1. For all treated groups, the significantly lower mite numbers, compared with the control group, were maintained through all assessments to Day 22. On Day 19, significant differences in the mean *O. sylviarum* vent counts were present between all groups.

These results demonstrate the potential for fluralaner to provide a substantial leap forward in the safe and effective management of northern fowl mite infestations. In addition, Maximum Residue Limits have now been approved in Europe at two single doses of 0.5 mg/kg, 7 days apart, for the treatment of poultry red mites, *Dermanyssus gallinae* (De Geer, 1778) [23]. Fluralaner can therefore be administered using standard medication equipment for drinking water to enhance the effectiveness of mite control measures in commercial poultry production. This offers the potential for reduced labor costs associated with spray or dust applications, reduced stress on birds, and reduced potential exposure to pesticides both of poultry-house workers and hens.

## Conclusions

A water-soluble formulation of fluralaner administered by oral gavage to laying hens on two occasions, with a one-week between-treatment interval, was effective in quickly controlling infestations with the northern fowl mite, *O. sylviarum.* Despite continued mite exposure by penning untreated, infested birds with fluralaner-treated birds, dose rates of 0.25, 0.5 and 1.0 mg/kg produced significant reductions in mean mite vent counts, relative to untreated controls, from Day 1 through Day 22. All dose rates maintained efficacy greater than 90% through Day 19, indicating that by providing an unprecedented level of northern fowl mite control fluralaner has the potential to improve the efficiency of poultry production, improve animal health and welfare, and eliminate the burden of topical pesticide application.

## Abbreviations

CVM: Center for Veterinary Medicine
FDA: United States Food and Drug Administration
GABA: γ-aminobutyric acid
IACUC: Institutional Animal Care and Use Committee
UGA: University of Georgia

## References

[1] Axtell RC, Arends JJ (1990). Ecology and management of arthropod pests of poultry. Annu Rev Entomol.

[2] Vezzoli G, King AJ, Mench JA. The effect of northern fowl mite (*Ornithonyssus sylviarum*) infestation on hen physiology, physical condition, and egg quality. Poult Sci. 2016;95:1042–9. 10.3382/ps/pew027 26944982

[3] Murillo AC, Chappell MA, Owen JP, Mullens BA. Northern fowl mite (*Ornithonyssus sylviarum*) effects on metabolism, body temperatures, skin condition, and egg production as a function of hen MHC haplotype. Poult Sci. 2016;95:2536–46. 10.3382/ps/pew175 27208153

[4] Mullens BA, Owen JP, Kuney DR, Szijj CE, Klingler KA. Temporal changes in distribution, prevalence and intensity of northern fowl mite (*Ornithonyssus sylviarum*) parasitism in commercial caged laying hens, with a comprehensive economic analysis of parasite impact. Vet Parasitol. 2009;160:116–33. 10.1016/j.vetpar.2008.10.076 19081198

[5] Lutsky I, Teichtahl H, Bar-Sela S (1984). Occupational asthma due to poultry mites. J Allergy Clin Immunol.

[6] Mullens BA, Kuney DR, Hinkle NC, Szijj CE (2004). Producer attitudes and control practices for northern fowl mites in southern California. J Appl Poult Res.

[7] Axtell RC, Dusbabek F, Bukva V (1991). Control of northern fowl mites on poultry. Modern acarology.

[8] Yazwinski TA, Tucker CA, Robins J, Powell J, Phillips M, Johnson Z, et al. Effectiveness of various acaricides in the treatment of naturally occurring *Ornithonyssus sylviarum* (northern fowl mite) infestations of chickens. J Appl Poult Res. 2005;14:265–8.

[9] Murillo AC, Mullens BA. A review of the biology, ecology, and control of the northern fowl mite, *Ornithonyssus sylviarum* (Acari: Macronyssidae). Vet Parasitol. 2017;246:30–7. 10.1016/j.vetpar.2017.09.002 28969777

[10] Ozoe Y, Asahi M, Ozoe F, Nakahira K, Mita T (2010). The antiparasitic isoxazoline A1443 is a potent blocker of insect ligand-gated chloride channels. Biochem Biophys Res Commun.

[11] Gassel M, Wolf C, Noack S, Williams H, Ilg T (2014). The novel isoxazoline ectoparasiticide fluralaner: selective inhibition of arthropod γ-aminobutyric acid- and L-glutamate-gated chloride channels and insecticidal/acaricidal activity. Insect Biochem Mol Biol.

[12] García-Reynaga P, Zhao C, Sarpong R, Casida JE (2013). New GABA/glutamate receptor target for [3H]isoxazoline insecticide. Chem Res Toxicol.

[13] Walther FM, Allan MJ, Roepke RK, Nuernberger MC (2014). Safety of fluralaner chewable tablets (Bravecto), a novel systemic antiparasitic drug, in dogs after oral administration. Parasit Vectors.

[14] Prohaczik A, Menge M, Huyghe B, Flochlay-Sigognault A, Le Traon G (2017). Safety of fluralaner oral solution, a novel systemic antiparasitic treatment for chickens, in laying hens after oral administration via drinking water. Parasit Vectors.

[15] Huyghe B, Le Traon G, Flochlay-Sigognault A (2017). Safety of fluralaner oral solution, a novel systemic poultry red mite treatment, for chicken breeders’ reproductive performances. Parasit Vectors.

[16] European Public Assessment Report (EPAR) for Exzolt - European Medicines Agency. http://www.ema.europa.eu/docs/en_GB/document_library/EPAR_-_Summary_for_the_public/veterinary/004344/WC500236956.pdf. Accessed 6 Feb 2018.

[17] United States Food and Drug Administration. Guidance for Industry Good Clinical Practice VICH GL9. http://www.fda.gov/downloads/AnimalVeterinary/GuidanceComplianceEnforcement/GuidanceforIndustry/UCM052417.pdf. Accessed 2 Feb 2017.

[18] Lemke LA, Collison CH. Evaluation of a visual sampling method used to estimate northern fowl mite, *Ornithonyssus sylviarum* (Acari: Macronyssidae), populations on caged laying hens. J Econ Entomol. 1985;78:1079–82. 10.1093/jee/78.5.1079 4056202

[19] Hofacre CL, Primm ND, Vance K, Goodwin MA, Brown J. Comparison of a lyophilized chicken-origin competitive exclusion culture, a lyophilized probiotic, and fresh turkey cecal material against *Salmonella* colonization. J Appl Poult Res. 2000;9:195–203.

[20] Romero C, Heredia R, Pineda J, Serrano JA, Mendoza GD, Trápala P (2016). Efficacy of fluralaner in 17 dogs with sarcoptic mange. Vet Dermatol.

[21] Taenzler J, Liebenberg J, Roepke RK, Frénais R, Heckeroth AR. Efficacy of fluralaner administered either orally or topically for the treatment of naturally acquired *Sarcoptes scabiei* var. *canis* infestation in dogs. Parasit Vectors. 2016;9:392. 10.1186/s13071-016-1670-7 PMC4937584 27387742

[22] Thomas E, Chiquet M, Sander B, Zschiesche E, Flochlay AS. Field efficacy and safety of fluralaner solution for administration in drinking water for the treatment of poultry red mite (*Dermanyssus gallinae*) infestations in commercial flocks in Europe. Parasit Vectors. 2017;10:457. 10.1186/s13071-017-2390-3 PMC5632831 28992814

[23] European Medicines Agency. European public MRL assessment report (EPMAR) Fluralaner (poultry) - EMA/CVMP/567262/2016 - 15 February 2017. http://www.ema.europa.eu/docs/en_GB/document_library/Maximum_Residue_Limits_-_Report/2017/02/WC500221753.pdf. Accessed 17 Apr 2017.

